# The E3 ligase SMURF1 stabilizes p27 *via* UbcH7 catalyzed K29-linked ubiquitin chains to promote cell migration SMURF1-UbcH7 K29 ubiquitination of p27 and cell migration

**DOI:** 10.1016/j.jbc.2024.105693

**Published:** 2024-02-01

**Authors:** Jasper Weinberg, Elizabeth Whitcomb, Andrew Bohm, Uday Kumar Chekkilla, Allen Taylor

**Affiliations:** Laboratory for Nutrition and Vision Research Human Nutrition Research Center on Aging Tufts University

**Keywords:** SMURF1, UBCH7 (UBE2L3), p27, ligase, ubiquitin conjugating enzyme, development, cell mobility

## Abstract

Ubiquitination is a key regulator of protein stability and function. The multifunctional protein p27 is known to be degraded by the proteasome following K48-linked ubiquitination. However, we recently reported that when the ubiquitin-conjugating enzyme UbcH7 (UBE2L3) is overexpressed, p27 is stabilized, and cell cycle is arrested in multiple diverse cell types including eye lens, retina, HEK-293, and HELA cells. However, the ubiquitin ligase associated with this stabilization of p27 remained a mystery. Starting with an *in vitro* ubiquitination screen, we identified RSP5 as the yeast E3 ligase partner of UbcH7 in the ubiquitination of p27. Screening of the homologous human NEDD4 family of E3 ligases revealed that SMURF1 but not its close homolog SMURF2, stabilizes p27 in cells. We found that SMURF1 ubiquitinates p27 with K29O but not K29R or K63O ubiquitin *in vitro*, demonstrating a strong preference for K29 chain formation. Consistent with SMURF1/UbcH7 stabilization of p27, we also found that SMURF1, UbcH7, and p27 promote cell migration, whereas knockdown of SMURF1 or UbcH7 reduces cell migration. We further demonstrated the colocalization of SMURF1/p27 and UbcH7/p27 at the leading edge of migrating cells. In sum, these results indicate that SMURF1 and UbcH7 work together to produce K29-linked ubiquitin chains on p27, resulting in the stabilization of p27 and promoting its cell-cycle independent function of regulating cell migration.

p27 has multiple crucial cellular roles. It is involved in cell proliferation, development, differentiation, and apoptosis and has distinct roles in the cytoplasm and nucleus (1). The wide variety of roles for p27 stems from its ability to form complexes with many different binding partners, including multiple CDKs, and proteins that regulate their activities and ubiquitin-dependent degradation (2). Misregulation and accumulation of p27 are associated with a variety of pathologies from human cancers to cataracts (3, 4, 5, 6, 7). While literature regarding p27 biochemistry in cell cycle regulation, proliferation, and degradation is well developed, far less is known about its roles in differentiation.

The attachment of ubiquitins to proteins requires a ubiquitin-activating enzyme, a ubiquitin-conjugating enzyme, and a ligase, respectively called E1, E2, and E3 enzymes. In most eukaryotic cells, there are a few E1s, a few dozen E2s, and hundreds of E3s. The vast combinatorial possibilities of these enzymes and other regulatory elements allow for the exquisite specificity of the ubiquitination system (8). The most common fate of ubiquitinated proteins is degradation by proteasomes (9).

p27 serves as a scaffold for the formation of multiple cyclin-CDK complexes. These complexes inhibit kinases that promote cell cycle progression, and proliferation, an activity associated with nuclear p27 (10). This activity is regulated by post-translational modifications including phosphorylation, export to the cytoplasm, and polyubiquitination using K48-linked ubiquitin. The formation of these polyubiquitinated species is catalyzed in the cytoplasm by the E2 Ubc3 and the E3 SCF^SKP2^, and p27 is degraded by the proteasome (11). In contrast, cytoplasmic p27 not marked for degradation can regulate the cytoskeleton, cell migration, apoptosis, autophagy, the epithelial to mesenchymal cell transition, and terminal differentiation processes such as removal of cell nuclei in lenses, this latter being required to complete formation of an optically clear eye lens (4, 5, 6, 7, 12, 13, 14, 15, 16, 17). Interestingly, stabilization of p27 by ubiquitination has only been reported once (18).

Importantly, the E2 enzyme UbcH7 promotes stabilization of p27, delaying entry into S phase of the cell cycle and affecting cell-cycle independent functions of p27, including cell migration (18, 19). UbcH7 can promote K29- and K63- linked ubiquitination of p27. Such ubiquitin linkages are not canonically associated with proteasomal degradation (18, 20). Therefore, we posited that UbcH7-catalyzed ubiquitination of p27 might be a mechanistic link to the accumulation of p27 and the phenotypes noted above.

But what is the E3 that cooperates with UbcH7 to attach ubiquitin to p27? There are three main classes of E3s: RING, HECT, and RBR. RING (Really Interesting New Gene) ligases position an E2 enzyme and substrate close to each other and facilitate transfer of the ubiquitin from the catalytic cysteine of the E2 to the acceptor lysine on the substrate (21). RBR (Ring Between Ring) ligases contain a RING1 domain which recruits an E2, an in-between-RINGs domain, and a RING2 domain, which contains a catalytic cysteine residue that accepts ubiquitin from the E2 and then transfers the ubiquitin to the substrate (22). HECT (Homologous to E6AP C-Terminus) domain ligases also have a catalytic cysteine residue that accepts ubiquitin from the E2, forming a thioester bond, before transferring the ubiquitin to the substrate (23). As UbcH7 is only able to discharge ubiquitin onto cysteine residues and not directly onto substrate lysine residues, it can only function with HECT and RBR E3 ligases which accept ubiquitin from the E2 and form a thioester bond between the E3 catalytic cysteine and ubiquitin (24). Consequently, we focused the search for the UbcH7-cooperating E3 on RBR and HECT classes of E3s (Table S1).

SMURF1 is a HECT ubiquitin E3 ligase of the NEDD4 family. It contains an N-terminal C2 domain, two WW domains, and a C-terminal HECT domain harboring its catalytic activity (23). Originally identified as a regulator of Smad protein stability in the TGF-B/BMP signaling pathway, SMURF1 has been shown to regulate the stability of a wide variety of targets in various pathways and disease states (25, 26). While most SMURF1-substrate interactions lead to degradation, SMURF1 is known to stabilize a subset of substrates *via* non-K48 linked ubiquitin chains. This includes the stabilization of PPARγ *via* K63-linked chains and the stabilization of axin *via* K29-linked chains (27, 28). However, a possible role for SMURF1 in stabilizing p27 *via* UbcH7-mediated ubiquitination has not been explored.

## Results

### *In vitro* ubiquitination: yeast extract

We previously observed that in the presence of UbcH7, p27 is stabilized rather than being degraded. This appeared to be due to the formation of K29 and K63 linked ubiquitin chains on p27, which are not usually associated with proteasomal degradation, but the identity of an E3 that cooperates with UbcH7 remained unknown. UbcH7 only functions with HECT and RBR class E3s, limiting the number of candidates to these classes of E3s. To identify an E3 enzyme that UbcH7 works with to stabilize p27, we exploited yeast, which has fewer candidate E3 enzymes (Table S1). Using the yeast deletion collection which has E3 knockouts in the same background, we screened mutants lacking the possible partners of UbcH7: HECT and RBR E3 (Fig. S1*G*). Western blots for ubiquitin in the p27 pulldowns from *in vitro* ubiquitination reactions containing extracts from which either HEL1, HUL5, UFD4, ITT1, or TOM1 were knocked out all revealed that p27 was still ubiquitinated (Fig. S1, *A*–*E*, compare the highlighted lanes 1 and 5). Since deletions of these HECT/RBR E3 ligases in yeast did not eliminate the ubiquitin signal in the p27 pulldowns, this implied that the E3 responsible for p27 ubiquitination was still present in the extract and the deleted E3 was not involved in the stabilization of p27. This left RSP5 as a candidate ligase for p27 ubiquitination and stabilization.

**Figure 1:**
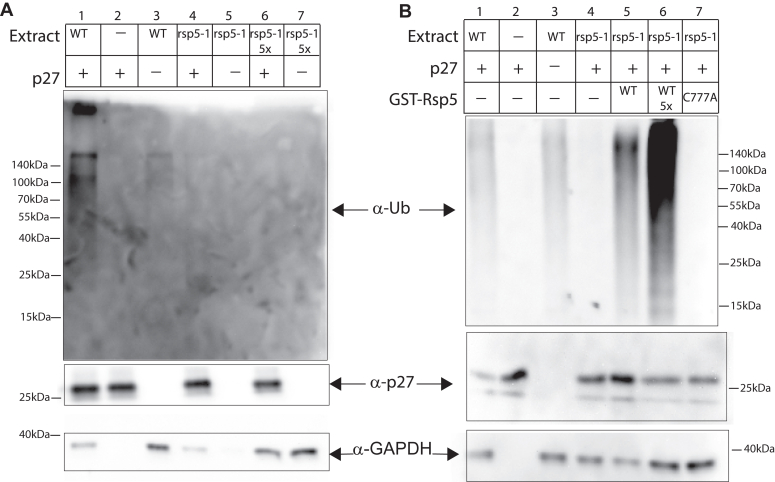
***In vitro* ubiquitination of p27 in yeast extract**. *In vitro* ubiquitination of p27 was carried out with yeast extracts with the indicated mutations, UbcH7, and His_6_-p27. His_6_-p27 was pulled down with Ni-NTA beads, and gels were blotted with anti-ubiquitin and anti-p27 antibodies. *A*, wild-type yeast extract shows strong ubiquitination of p27 (lane 1) whereas the no p27 (lane 3) and no extract (lane 2) controls show little ubiquitination. The addition of rsp5-1 mutant extract does not promote p27 ubiquitination (lane 4), even with the addition of excess extract (lane 6). The *lower panel* shows equal loading of p27 in lanes 1, 2 4, and 6. *B*, as in *A*, WT but not rsp5-1 mutant extract supports p27 ubiquitination (lane 1 vs lane 4). The addition of purified Rsp5 promotes p27 ubiquitination in a dose-dependent manner (lanes 4, 5, 6), while the addition of a catalytically dead mutant Rsp5 does not support p27 ubiquitination (lane 7). The *lower panel* shows p27 loading. The *bottom panel* shows GAPDH loading control.

With RSP5 present, ubiquitination of p27 was also observed (Fig. 1, *A* and *B* lanes 1 *versus* 2). However, in contrast with results for the other five HECT/RBR E3 ligases, a temperature-sensitive mutation of the HECT E3 ligase RSP5 (rsp5-1) completely ablated the ubiquitin signal in the pulldowns (Fig. 1, *A* and *B* compare lane 1 *versus* 4). The addition of more mutant rsp5-1 extract did not increase ubiquitination demonstrating that no other E3 catalyzes the p27 ubiquitination observed (Fig. 1*A*, lanes 4 and 6 *versus* lane 1). To test the hypothesis that RSP5 is the E3 that catalyzes ubiquitination of p27 we added increasing amounts of purified Rsp5p to the reaction. This increased the ubiquitinated p27 in a dose-dependent fashion (Fig. 1*B*, lanes 5, 6). In contrast, the addition of inactive mutant Rsp5p did not catalyze the ubiquitination of p27 (Fig. 1*B* lane 7). These data indicate that RSP5 is the yeast E3 ligase that works with UbcH7 to ubiquitinate p27.

### SMURF1 and UbcH7 stabilize p27

**Figure 2:**
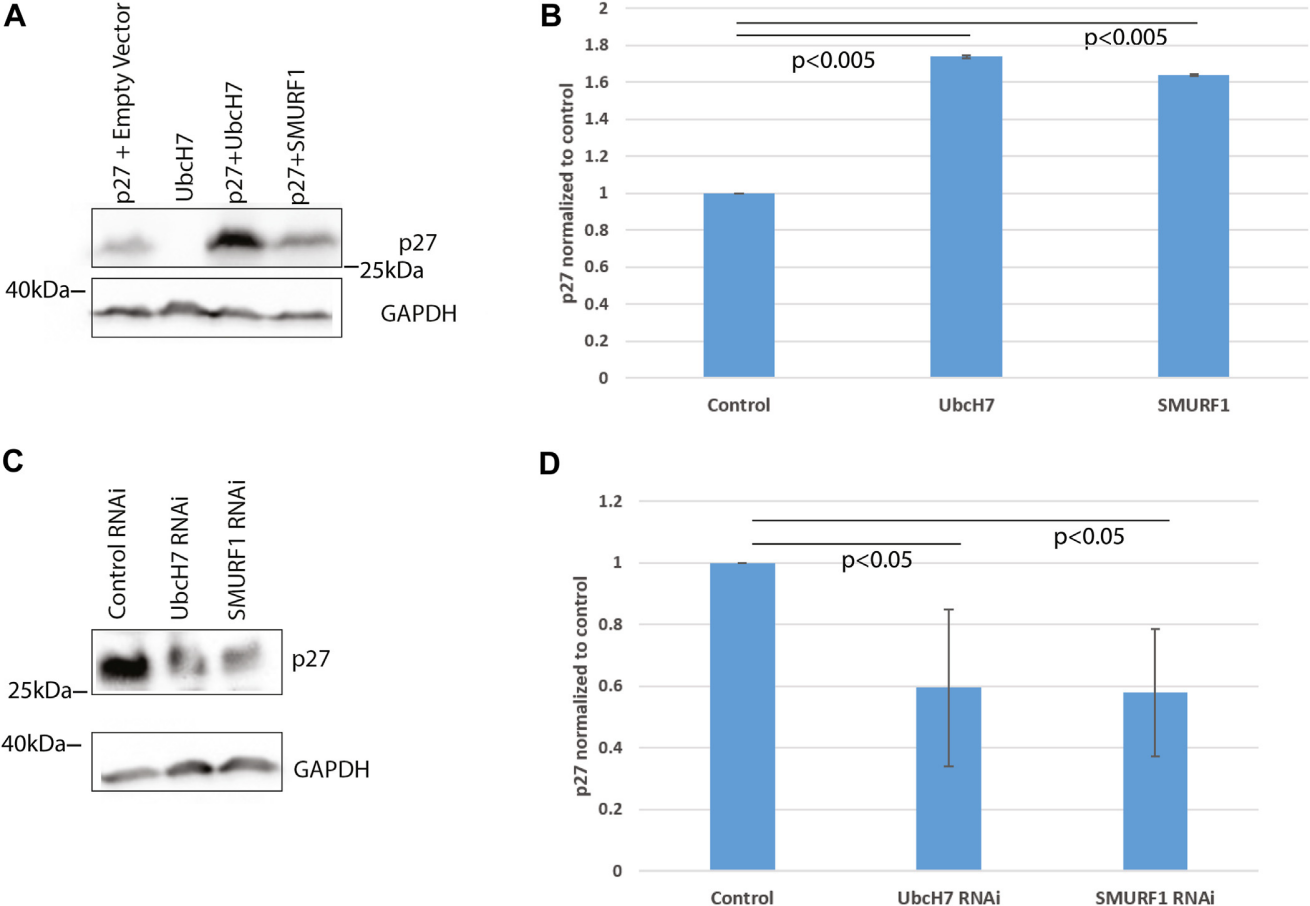
**SMURF1 and UbcH7 control p27 levels in cell culture.***A*, UbcH7 or SMURF1 were overexpressed and the level of p27 was determined by Western blotting. *B*, quantification of at least three independent overexpression experiments. *C*, HeLa cells were transfected with RNAi to reduce the levels of UbcH7 or SMURF1, both of which decreased the levels of p27. *D*, quantification of at least three independent experiments is shown.

**Figure S2:**
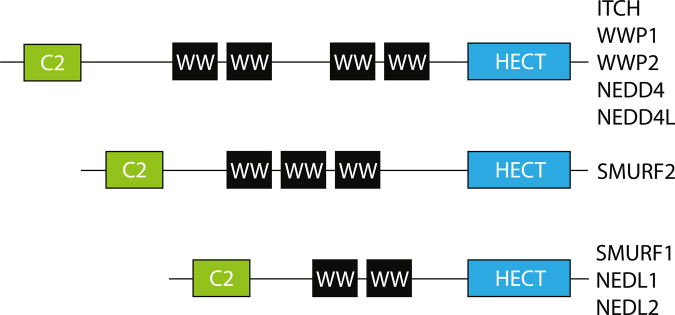
The Nedd4 family of HECT domain E3 ubiquitin ligases has nine members. All family members have a N-terminal C2 domain and a C-terminal HECT domain which contains the catalytic activity. The central region of the proteins have a variable number of WW domains that interact with other proteins, often recruiting substrates. ITCH, WWP1, WWP2, NEDD4, and NEDD4L have 4 WW domains. SMURF2 has 3 WW domains. SMURF1, NEDL1, and NEDL2 have 2 WW domains. These diagrams are representative schematics and are not to scale.

**Figure S3:**
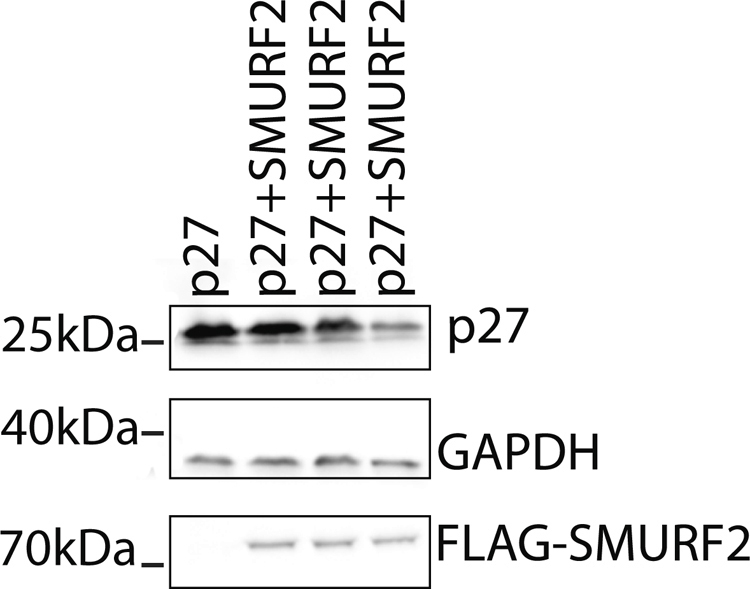
HELA cells were transfected with p27 and SMURF2 plasmids, and Western blots were run of the extracts. Increasing the SMURF2 levels decreases the p27 levels, as previously reported (compare lanes 2-4 versus 1).

**Figure S4:**
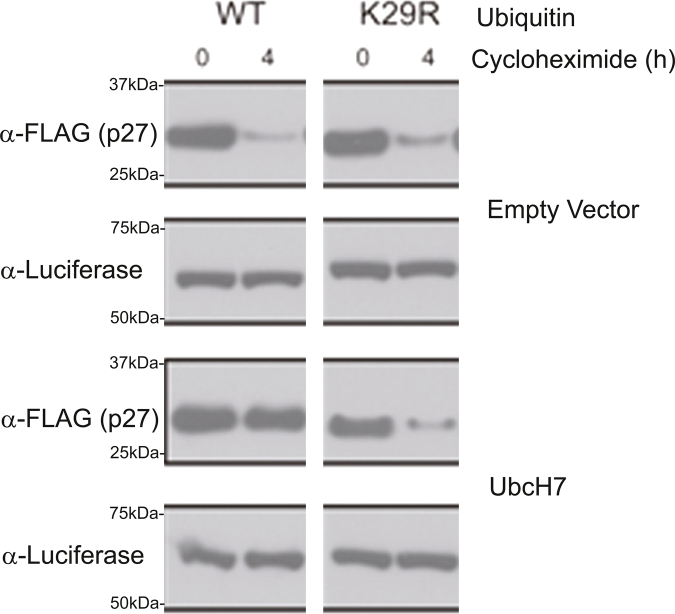
HEK-293 cells were transfected with FLAG-p27, either empty vector or UbcH7, and either wild-type or K29R ubiquitin. A 4 h cycloheximide chase was used to assess protein stability. Expression of UbcH7 stabilizes p27 compared to the empty vector. Expression of K29R prevents the UbcH7-dependent p27 stabilization. Adapted from Figure 5 of Whitcomb et al (18).

What is the mammalian E3 that cooperates with ubcH7 to stabilize p27? RSP5 is the yeast homolog of the mammalian NEDD4 family of E3 ligases, which consists of NEDL1/2, NEDD4, NEDD4L, SMURF1/2, WWP1/2 and ITCH (Fig. S2). Previous literature identified WWP1/2, SMURF2, and NEDD4 as E3s that promote degradation of p27 (29, 30, 31, 32). To determine which of these E3s is involved in p27 ubiquitination we tested each of the members of the NEDD4 family for their effects on p27 levels, focusing on those not previously identified as promoting p27 degradation. As with UbcH7, overexpression of SMURF1 increased p27 levels (Fig. 2, *A* and *B*) while overexpression of the closely related homolog SMURF2 decreased p27 levels as previously reported (Fig. S3), (29). Conversely RNAi knockdown of UbcH7 or SMURF1 decreased p27 levels (Fig. 2, *C* and *D*). This demonstrates that, like UbcH7, SMURF1 works to stabilize p27, a function that is distinct from its close homolog SMURF2. Overexpression of other NEDD4 family members did not stabilize p27 (data not shown). A cycloheximide chase experiment showed that overexpression of UbcH7 stabilizes FLAG-p27 compared to expression of the empty vector (Fig. S4 WT column). The UbcH7-dependent stabilization is blocked by the expression of K29R ubiquitin, demonstrating that K29 chains are required for UbcH7-mediated p27 stabilization (Fig. S4 K29R column).

### AlphaFold

**Figure 3:**
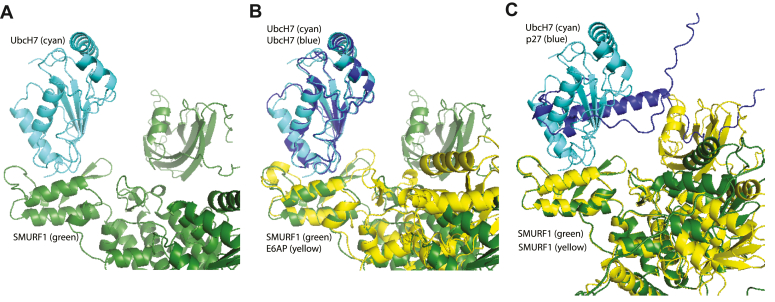
**AlphaFold predicts UbcH7 and p27 interactions with SMURF1.***A*, alphaFold predicted an interaction between UbcH7 (*cyan*) and the HECT domain of SMURF1 (*green*). *B*, aligning the predicted UbcH7-SMURF1 structure (*cyan* and *green* respectively) with the crystal structure of UbcH7 (*blue*) and the HECT domain of E6AP (*yellow*) (PDB: 1C4Z) shows that the two complexes overlap significantly, particularly at the interaction site. *C*, the predicted structure of p27 (*blue*) with SMURF1 (*green*) is shown superimposed on the predicted structure of UbcH7 (*cyan*) with SMURF1 (*yellow*), revealing that UbcH7 and p27 are predicted to interact with the same domain of SMURF1, precluding the formation of a ternary complex.

To gain more insight into the interactions between SMURF1, UbcH7, and p27, we used the powerful AI structural prediction tool AlphaFold to predict pairwise interactions between SMURF1 and UbcH7 or p27 (33, 34). Consistent with earlier NMR studies, the algorithm predicted an intramolecular interaction between the C2 and HECT domains in SMURF (35). AlphaFold also predicted an interaction between the HECT domain of SMURF1 and UbcH7 (Fig. 3*A*). The same intermolecular interactions are seen in all 25 of the top output models (not shown). The molecular details of the predicted UbcH7-SMURF1 interaction are very similar to those seen in the solved crystal structure of E6AP-UbcH7 obtained using x-ray diffraction (Fig. 3*B*) (36). Outside of the interacting region, the AlphaFold model differs significantly from the structure of UbcH7 and E6AP (Fig. 3*B* right side).

AlphaFold also predicted an interaction between p27 and the HECT domain of SMURF1 (Fig. 3*C*). According to AlphaFold, p27 is mostly unstructured with only two helices that interact with the HECT domain of SMURF1. We then asked AlphaFold to generate a model of the ternary complex between SMURF1, p27, and UbcH7. AlphaFold did not predict the formation of a ternary complex. This is likely because p27 and UbcH7 are predicted by AlphaFold to interact with the same region of SMURF1 (Fig. 3*C*).

**Figure S5:**
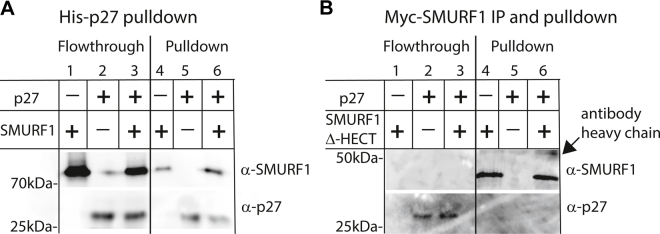
*A*, His-p27 and FLAG-SMURF1 were allowed to interact, and then the p27 was pulled down with Ni-NTA beads. The flowthrough and bead bound proteins were analyzed by Western blot. His-p27 was able to pull down SMURF1. *B*, Myc-SMURF1 -HECT was immunoprecipitated from HELA cells using anti-Myc antibody and protein G beads. His-p27 was then incubated with the beads, and Western blots were used to analyze the bead bound proteins after washing. His-p27 did not interact with the truncated SMURF1.

To validate the AlphaFold predictions we performed pulldowns to interrogate the interaction between p27 and SMURF1. His-p27 on nickel beads co-purified with full-length SMURF1 (Fig. S5*A* lane 6). To test the predicted importance of the HECT domain in this interaction, we immunoprecipitated Myc-SMURF1 lacking the HECT domain from HELA cells, then added the same His-p27 as before. The His-p27 did not interact with the bead-bound SMURF1 -HECT (Fig. S5*B* lane 6).

### *In vitro* ubiquitination of p27 by SMURF1 and UbcH7 produces K29-linked chains

**Figure 4:**
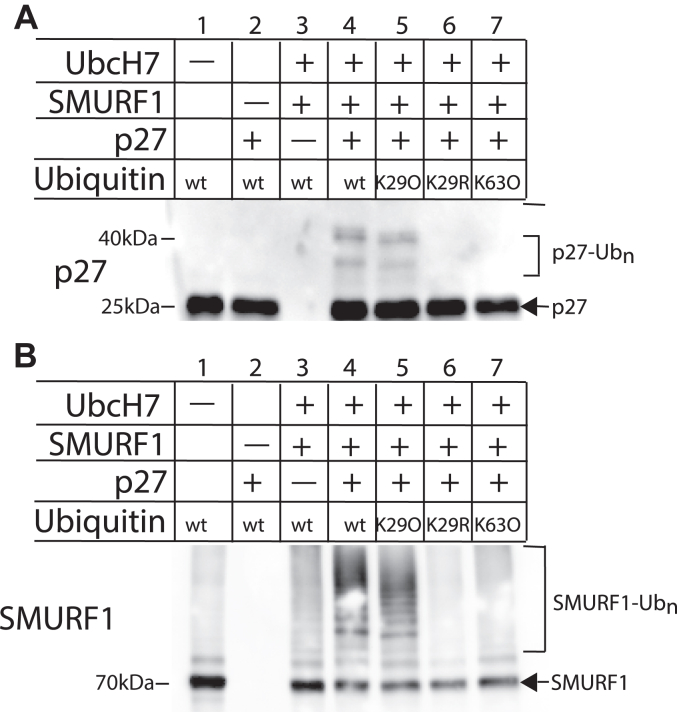
**UbcH7-SMURF1 prefers conjugating K29 ubiquitin to p27**. Ubiquitination of p27 was carried out in a reconstituted system with Ube1, UbcH7, SMURF1, His_6_-p27, ATP regeneration system, and buffer. Reactions were run on SDS-PAGE gels and probed with anti-SMURF1 and anti-p27 antibodies. *A*, the p27 immunoblot shows p27 is ubiquitinated by SMURF1 with wild-type (lane 4 *vs.* lane 2) and K29O ubiquitin (lane 5 *vs.* lane 2) but not K29R (lane 6) or K63O ubiquitin (lane 7). *B*, the immunoblot for SMURF1 shows SMURF1 auto-ubiquitination with wild-type (lanes 3 and 4), K29O (lane 5), and, to a lesser extent, K63O ubiquitin (lane 7). Autoubiquitination is more extensive with p27 present (Lane 4 *versus* 3).

To determine if there is a functional interaction between SMURF1, UbcH7, and p27, we performed ubiquitination reactions using purified components. SMURF1 ubiquitinated p27 with wild-type ubiquitin in the presence of UbcH7 (Fig. 4*A* lane 4). This did not occur in the absence of UbcH7 or SMURF1 (Fig. 4 lane 1 and 2, respectively). These findings confirm that UbcH7 and SMURF1 together catalyze the ubiquitination of p27. Interestingly, p27 ubiquitination was also observed in reactions with a ubiquitin variant that has only K29, with all other lysine residing mutated to arginine (K29O) (Fig. 4*A* lane 5). K29R ubiquitin, which does not support the formation of K29 chains, shows no p27 ubiquitination (Fig. 4*A* lane 6). SMURF1 also showed no p27 ubiquitination with K63O ubiquitin (Fig. 4*A* lane 7). Overall, these results demonstrate that SMURF1, together with UbcH7, is capable of forming K29 ubiquitin chains on p27.

We observed SMURF1 autoubiquitination that increased in the presence of UbcH7, as reported previously (Fig. 4*B* compare lanes 1 and 3) (37). Curiously, the addition of p27 increased UbcH7-catalyzed SMURF1 autoubiquitination (Fig. 4*B* compare lanes 3 and 4). SMURF1 autoubiquitination was affected by the ubiquitin variant present in the reaction. SMURF1 showed strong autoubiquitination activity with wild-type ubiquitin (Fig. 4*B*, lane 4) and with K29O ubiquitin (Fig. 4*B* lane 5), but only weak autoubiquitination with K63O ubiquitin (Fig. 4*B*, lane 7). There was no detectable SMURF1 autoubiquitination with K29R ubiquitin (Fig. 4*B*, lane 6). These findings suggest that the UbcH7/SMURF1 pairing is most efficient at auto-ubiquitination of SMURF1 using K29 on ubiquitin.

### Functional ramifications of an UbcH7-SMURF1-p27 interaction

**Figure 5:**
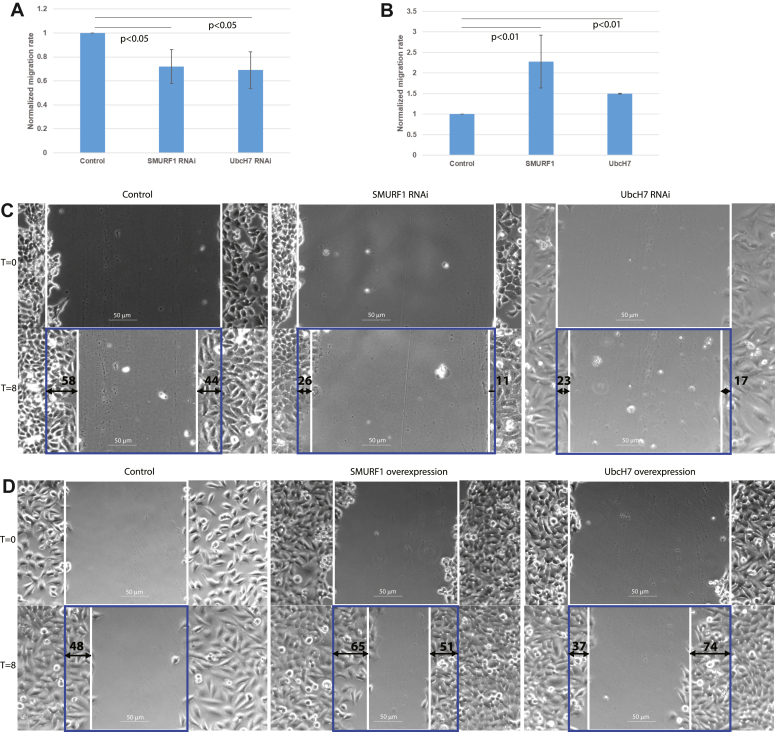
**Migration assays**. Migration assays were performed by scratching a monolayer of cells with a P20 pipet tip. The rate at which the scratch closed was measured at 8 h after injury. *A*, knockdown of SMURF1 or UbcH7 by RNAi decreased the rate of migration. *B*, overexpression of UbcH7 or SMURF1 increases the rate of migration. *C*, migration is slower when SMURF1 or UbcH7 are diminished. Example images of cells migrating into a scratch for an RNAi experiment. Time = 0 (*upper panels*) and time = 8 h (*lower panels*) after scratch are shown. *D*, migration is faster when SMURF1 or UbcH7 are overexpressed. Example images from an overexpression experiment showing increased migration rate for SMURF1 and UbcH7 expressing cells. *White boxes mark* the scratched area free of cells, *blue boxes mark* the extent of the initial scratch. Numbers and *arrows*, in *black*, indicate the change in the width of the scratch on either side. For calculations, these numbers are summed and averaged at three equidistant points down the scratch. Graphs are an average of at least three independent experiments.

**Figure S6:**
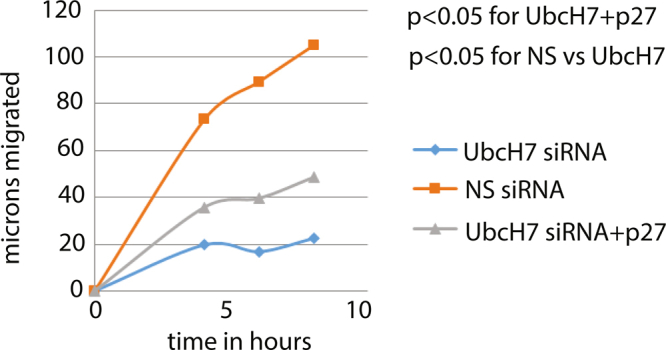
RNAi knockdown of UbcH7 reduces the rate of cell migration. Overexpression of p27 in UbcH7 knockdown cells increases the rate of cell migration, partially rescuing the defect.

Cytoplasmic p27 is known to affect cell migration *via* its interaction with cytoskeletal regulators such as RhoA (12). In order to demonstrate a functional role for the SMURF1 stabilization of p27 we assessed the impact of SMURF1 and UbcH7 on cell migration. RNAi knockdown of UbcH7 or SMURF1, consistent with a decrease in p27, decreased the rate of cell migration as measured by a scratch assay (Fig. 5*A*). We corroborated these findings by showing that expression of p27 increased cell migration in cells in which UbcH7 was knocked down by RNAi (Fig. S6). Conversely, overexpression of UbcH7 or SMURF1 increased the rate of cell migration (Fig. 5*B*). Typical micrographs of migration assays are shown in Figure 5, *C* and *D*. These findings indicate that SMURF1, UbcH7, and p27 are part of a pathway that controls cell migration.

### Colocalization of SMURF1, UbcH7, and p27

**Figure 6:**
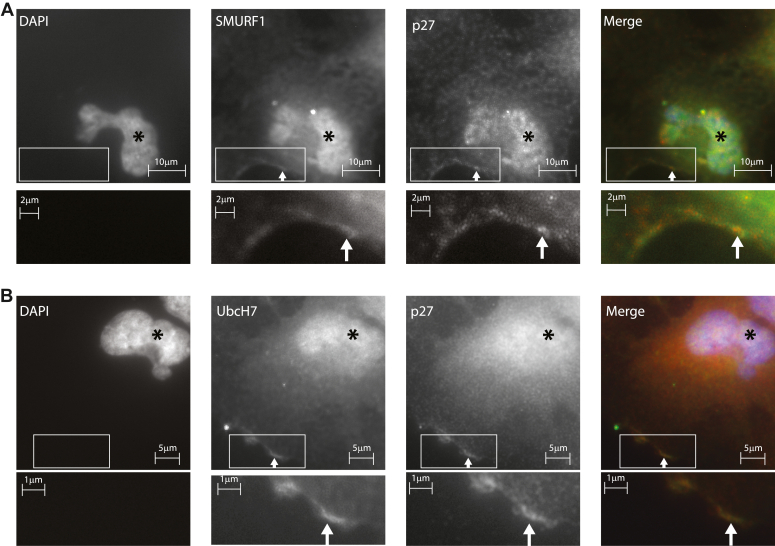
**Colocalization of SMURF1-p27 and UbcH7-p27**. Immunofluorescence staining was performed on migrating HLEC cells after a scratch disrupted the monolayer. *A*, colocalization is evident between SMURF1 and p27 both in the nucleus and in the cytoplasm at the cell periphery at the front of migrating cells (*arrows*). *Lower panels* are enlargements of the *boxed area*. *B*, colocalization is shown between UbcH7 and p27 both in the nucleus and in the cytoplasm at the cell periphery (*arrows*). See Fig. S7 for secondary antibody only controls.

We wondered if we could colocalize SMURF1, UbcH7, and p27, especially in the cytoplasm where p27 exerts its influence on migration *via* the cytoskeleton (12). Staining of human lens epithelial cells (HLECs) after disrupting a monolayer *via* a scratch demonstrates colocalization of SMURF1 and p27 in the nucleus (Fig. 6*A*, asterisk) and in the cytoplasm at leading edge of migrating cells (Fig. 6*A*, arrows highlight cytoplasmic colocalization). UbcH7 and p27 also show this colocalization in both the nucleus and at the leading edge (Fig. 6*B*, asterisks and arrows respectively). These findings are consistent with the influence of the UbcH7, SMURF1, p27 triplet on cell migration, as noted above.

## Discussion

The canonical function of p27 is as a regulator of the cell cycle. That requires its phosphorylation and subsequent ubiquitination and degradation which allows for progression through the cell cycle and cell proliferation. p27 also regulates cell migration and multiple other physiological functions, so it is not surprising that the regulation of p27 is complex and varied. Herein we explored a different mode of ubiquitin regulation for p27 from the canonical proteasome degradation. We find that K29 ubiquitination of p27 by the UbcH7-SMURF1 couple stabilizes p27 and promotes cell migration. This information augments current understanding of the biology of p27 and the ubiquitination pathways involved in that regulation.

Our prior research indicated that the E2 enzyme UbcH7 promotes p27 stability, but the E3 ligase working with UbcH7 was unknown. Substrate ubiquitination using K29 and K63 chains does not lead to proteolysis. Rather, such ubiquitination regulates protein-protein interactions. K63 chains in particular are known to promote the formation of signaling complexes involved in pathways from DNA damage response to immune regulation as well as regulating endocytosis (20). K29 chains are less well studied, but SMURF1-catalyzed K29 ubiquitination blocks Axin’s interactions with Wnt receptors, thus decreasing Wnt signaling (28).

Because they have fewer E3s and no UbcH7, yeast provided a useful system to screen for the E3 that can work with UbcH7 to stabilize p27. We first identified the relevant yeast E3 ligase, Rsp5p. Whereas extract from yeast lacking various E3s showed p27 ubiquitination, only extract from RSP5 mutant cells did not support p27 ubiquitination. In confirmation of a role for Rsp5p in the ubiquitination of p27, ubiquitination of p27 could be rescued by the addition of wild-type but not catalytically dead Rsp5p (Fig. 1).

RSP5 is the yeast homolog of the mammalian Nedd4 family of E3 ligases. In order to find the mammalian E3 that works with UbcH7 to stabilize p27, we tested the effects of overexpressing individual Nedd4 family members on p27 levels (Figs. 2 and S3). Overexpression of SMURF1 stabilized p27 while knocking down SMURF1 reduced p27 levels. This suggests that SMURF1 is the E3 ligase that works with UbcH7 to stabilize p27. Other NEDD4 family members ITCH, WWP1, WWP2, NEDD4, NEDD4L, NEDL1, NEDL2, and SMURF2 did not participate in p27 stabilization. *In vitro* ubiquitination reactions confirmed that SMURF1 was capable of modifying p27 with K29 chains, consistent with a role in stabilizing p27.

The hypothesis that these proteins are working together is further supported by our AlphaFold modeling which predicts an interaction between UbcH7 and the HECT domain of SMURF1 that is similar to the solved structure of UbcH7-E6AP. AlphaFold also predicted an interaction between the HECT domain of SMURF1 and p27, one that overlaps with the UbcH7-SMURF1 interaction. This is consistent with SMURF1 binding to UbcH7 and p27 sequentially, receiving ubiquitin from the E2 and then transferring it to the p27. On the other hand, NEDD4 family E3s, such as SMURF1, often recruit substrates *via* their WW domains, which interact with a variety of phosphopeptides and proline-rich motifs, both of which p27 contains (38, 39). While AlphaFold did not predict p27 binding to a WW domain or a ternary complex between p27, SMURF1, and UbcH7, such an interaction may be difficult to predict due to p27’s intrinsically unstructured nature, or there may be another cofactor that assists in bringing p27, SMURF1, and p27 together.

We next established a functional role for SMURF1 and UbcH7 in regulating p27 stability by demonstrating that SMURF1 or UbcH7 knockdown reduced the speed of cell migration while overexpression of either protein increased the speed of cell migration (Fig. 5). Further evidence for a functional relationship between UbcH7, SMURF1, and p27 comes from our demonstration of colocalization of these proteins at the leading edge of migrating cells (Fig. 6).

Overall, we elucidate another facet of the regulation of the multifunctional protein p27, a stabilizing K29-linked ubiquitination by UbcH7 and SMURF1 working together. This modification is functionally relevant as disrupting either the E2 or the E3 impacts cell migration. The SMURF1-UbcH7 axis therefore ubiquitinates p27 with K29-linked ubiquitin chains, preventing its cytoplasmic degradation and thus promoting other functions, such as enhancing cell migration. This regulation might be by populating K48 ubiquitination sites that would lead to degradation with K29 chains, or by K29 ubiquitin chains recruiting different binding partners than those that promote p27 degradation.

This information may be medically relevant as well. We previously observed that multiple congenital cataract models show stabilization of p27 (5, 7). These cataract models also show delays in the unidirectional process of Cdk1-and p27-directed lens fiber cell denucleation, a process required to establish a clear and functional eye lens. It will be interesting to determine the specific role of K29-linked ubiquitination of p27 in this process. It will also be interesting to explore whether this pairing of UbcH7 and SMURF1 is the functional unit for other proteins known to be stabilized by either UbcH7, such as p53 and p-PTEN, or stabilized by SMURF1, such as axin (28).

## Experimental procedures

### Cell culture

Yeast strains from the deletion collection were a gift from Claire Moore (Tufts University), rsp5-1 was a gift from Fred Winston (Harvard Medical School). Yeast strains were grown in YPD (Sigma). Yeast were grown to log phase, frozen in liquid nitrogen, and thawed as needed. The extract was prepared fresh by bead beating in 20 mM Tris pH 7.9, 50 mM KCl, 0.5 mM DTT, 10% glycerol, with PMSF and N-ethylmaleimide (Sigma).

Hela and human lens epithelial cells were maintained in DMEM supplemented with 10% FBS and penicillin/streptomycin. Transfections were carried out in OptiMEM medium using Lipofectamine 2000 or Lipofectamine 3000 according to the manufacturer’s directions. Smurf1 and Smurf2 RNAi were from Dharmacon, UbcH7 RNAi from Qiagen. Extracts were prepared in RIPA buffer (Thermo Scientific) supplemented with protease inhibitor cocktail (Sigma) and N-ethylmaleimide (Sigma). Nedd4 family ligase expression vectors were a kind gift from Dr Wesley Sundquist (University of Utah). GST-Rsp5 and GST-Rsp5 C777A were a gift from Dr Joseph Reese (Penn State).

### Migration assays

For wound healing assays cells were transfected in 24 well plates according to the manufacturer’s directions, and a line was drawn through the monolayer 48 h after transfection with a P20 pipet tip. The media was changed to remove loose cells and the scratch was imaged at 10× using a Zeiss Axiovert 200 at T = 0 and T = 8. The width of the scratch was measured in ImageJ (NIH) at three locations per image and averaged.

### *In vitro* ubiquitination

For in extract ubiquitination: (His)_6_-p27 was bound to Ni-NTA beads (Qiagen) for 1 h, washed with 20 mM Tris pH 7.5 10 mM imidazole, beads were blocked with BSA for 1 h, and washed as before. Reactions were assembled containing 5× Buffer (200 mM Tris pH 7.6, 25 mM MgCl_2_, 10 mM DTT), MG132, ATP, creatine phosphokinase, creatine phosphate, ubiquitin, yeast extract, and UbcH7. Reactions were stopped after 90 min by the addition of Laemmli sample buffer and run on 10.5% SDS-PAGE gels. Antibodies used were p27 (AF2256 R&D Systems), ubiquitin (made in house), blots were developed with ECL (Prometheus ProSignal Femto, Genesee Scientific) and imaged using a FluorChemQ MultiImage III (Alpha Innotech).

For reconstituted *in vitro* ubiquitination: reactions were assembled in buffer (final concentration: 40 mM Tris pH7.6, 5 mM MgCl2, 2 mM DTT) with ATP, creatine phosphokinase, creatine phosphate, ubiquitin, Ube1, UbcH7, p27, and SMURF1. Reactions were stopped after 90 min by the addition of Laemmli sample buffer and run on 10.5% SDS-PAGE gels. Antibodies used were SMURF1 (sc-100616 Santa Cruz Biotechnology), p27 (AF2256 R&D Systems), ubiquitin (made in house), blots were developed with ECL (Prometheus ProSignal Femto Genesee Scientific) and imaged using a FluorChemQ MultiImage III (Alpha Innotech).

### Pulldowns

For the His-p27 pulldown: FLAG-SMURF1 (BPS Bioscience) was rocked at 4°C with (His)_6_-p27 (purified in house) and Ni-NTA beads (Qiagen) (blocked with BSA for 1 h) for 2 h in 1× ubiquitination buffer (40 mM Tris pH 7.6, 5 mM MgCl_2_, 2 mM DTT). Beads were pelleted in a microfuge and washed 5×. Flowthrough and bead-bound proteins fractions were run on 10.5% SDS-PAGE gels. Antibodies used were SMURF1 (sc-100616 Santa Cruz Biotechnology) and p27 (AF2256 R&D Systems).

For the Myc-SMURF1 -HECT pulldown: Myc-SMURF1 -HECT (Addgene 13677) was transfected into HELA cells using Lipofectamine 3000 as per the manufacturer’s instructions. Cells were lysed in RIPA buffer (Thermo Scientific) and SMURF1 was immunoprecipitated using an anti-Myc antibody (9E10 Santa Cruz Biotechnology) and protein G agarose (Roche). Beads were then rotated with (His)_6_-p27 (purified in house) in 1× ubiquitination buffer (40 mM Tris pH 7.6, 5 mM MgCl_2_, 2 mM DTT) at 4 °C for 2 h. Beads were pelleted in a microfuge and washed 5×. Flowthrough and bead-bound proteins fractions were run on 10.5% SDS-PAGE gels. Antibodies used were SMURF1 (sc-100616 Santa Cruz Biotechnology) and p27 (AF2256 R&D Systems).

### Immunofluorescence

HLEC were grown on coverslips to confluence. Coverslips were fixed in 4% paraformaldehyde and rinsed in PBS. Cells were permeablilzed in PBS with 0.5%Tween and blocked with 5% normal donkey serum. Antibody to UbcH7 (610852 BD Biosciences). Secondary antibodies Alexa 488 anti-goat (Jackson Immunoresearch) Cy3 anti-mouse (Jackson Immunoresearch).

### AlphaFold and molecular graphics

AlphaFold version 2.2.0 was run on the Tufts High Performance Computing Cluster using the multimer option. Molecular graphics figures were generated using the Open Source version of PyMOL released by Schrödenger.

## Data availability

All data are contained within this paper.

## Supporting information

This article contains supporting information (18).

## Conflict of interest

The authors declare that they have no known competing financial interests or personal relationships that could have appeared to influence the work reported in this paper.

## Figures and Tables

**Figure S1:**
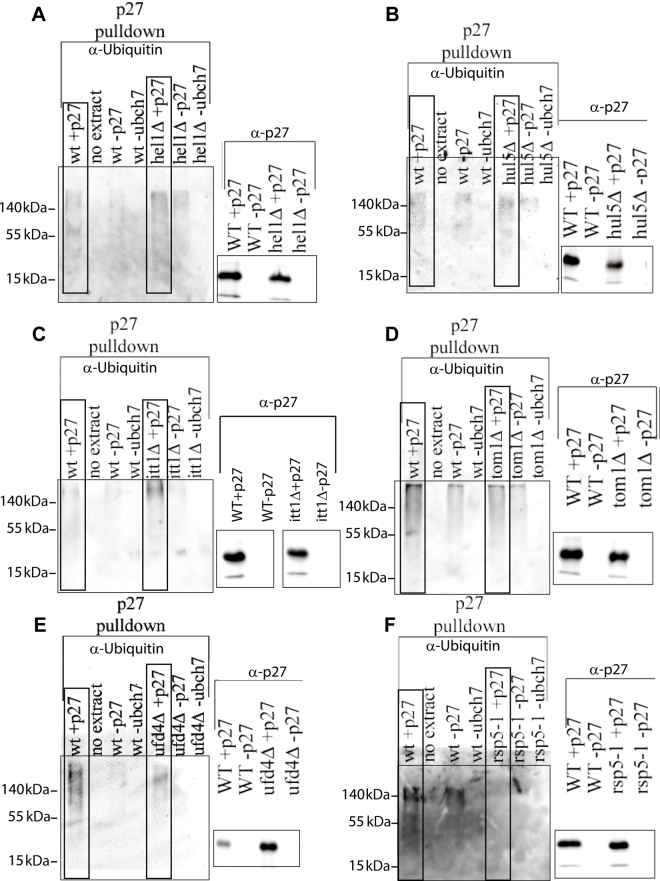
Representative blots of p27 pulldowns from ubiquitination assays run in yeast extract from the indicated E3 ligase knockout strains. *A*–*E*, ubiquitination of p27 is seen in the knockout strain (lane 5), demonstrating that those ligases are not responsible for p27 ubiquitination in yeast extract, while in *F*, lane 5 has no ubiquitin signal, demonstrating that RSP5 is the ligase responsible for p27 ubiquitination. The p27 blots, run with the same *in vitro* reactions in parallel experiments, demonstrate that the reduction in ubiquitin signal in *F* is not due to changes in p27 loading.

**Figure S1G:**
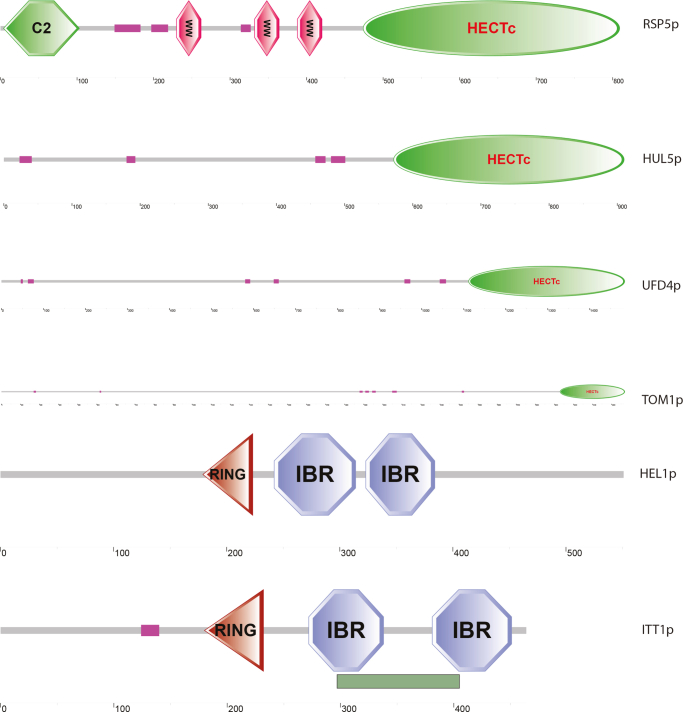
*G*, SMART domain analyses of the yeast HECT and RBR ligases (http://smart.embl-heidelberg.de/). RSP5 is a NEDD4 family homolog containing an N-terminal C2 domain, three WW domains, and a C-terminal HECT domain. HUL5, UFD4, and TOM1 are all HECT domain ligases. HEL1 and ITT1 are RBR ligases with RING and IBR domains.

**Figure S7:**
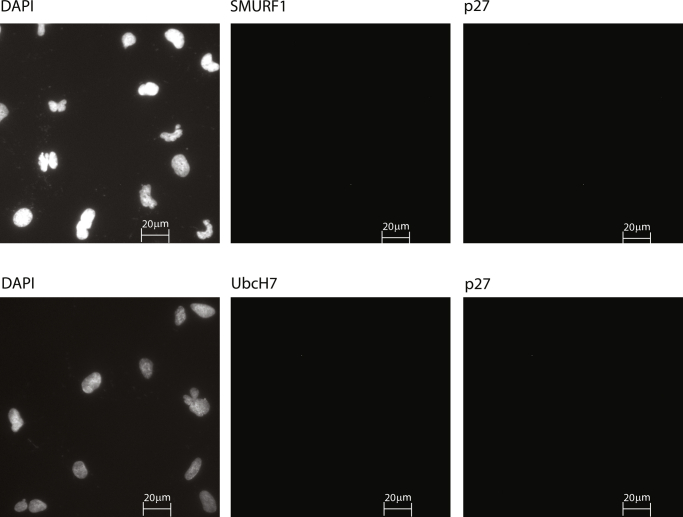
Control slides stained with secondary antibody alone are shown, demonstrating no significant background staining for any antibodies used.

**Table S1:**
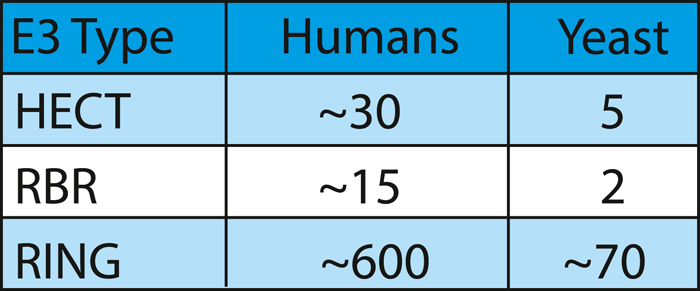
While the vast majority of E3 ligases in both systems are RING type ligases, yeast has many fewer of each type of ligase (HECT, RBR, and RING) than humans.
